# The impact of car ownership and public transport usage on cancer screening coverage: Empirical evidence using a spatial analysis in England

**DOI:** 10.1016/j.jtrangeo.2016.08.012

**Published:** 2016-10

**Authors:** Chao Wang

**Affiliations:** Centre for Cancer Prevention, Wolfson Institute of Preventive Medicine, Queen Mary University of London, Charterhouse Square, London EC1M 6BQ, United Kingdom

**Keywords:** Car ownership, Public transport, Cancer screening, Spatial analysis, Spatial correlation, England

## Abstract

A spatial analysis has been conducted in England, with the aim to examine the impact of car ownership and public transport usage on breast and cervical cancer screening coverage. District-level cancer screening coverage data (in proportions) and UK census data have been collected and linked. Their effects on cancer screening coverage were modelled by using both non-spatial and spatial models to control for spatial correlation.

Significant spatial correlation has been observed and thus spatial model is preferred. It is found that increased car ownership is significantly associated with improved breast and cervical cancer screening coverage. Public transport usage is inversely associated with breast cancer screening coverage; but positively associated with cervical cancer screening. An area with higher median age is associated with higher screening coverage. The effects of other socio-economic factors such as deprivation and economic activity have also been explored with expected results. Some regional differences have been observed, possibly due to unobserved factors.

Relevant transport and public health policies are thus required for improved coverage. While restricting access to cars may lead to various benefits in public health, it may also result in worse cancer screening uptake. It is thus recommended that careful consideration should be taken before implementing policy interventions.

## Highlights

• This paper examines the impact of car ownership on cancer screening coverage.
• It also explores the impact of public transport usage on cancer screening.
• A spatial district-level analysis has been conducted in England.
• Spatial correlation has been explicitly controlled for.
• Results show strong positive impact of car ownership on screening coverage.

## 1 Introduction

Screening is an important tool to detect cancer at early stage and is estimated to save thousands of lives in England every year (Public Health Outcomes Framework, 2014). While the benefit of screening is significant, there are various barriers preventing people attending the screening; and thus the screening uptake can differ significantly from one area to another.

A number of factors have been identified to have an impact on screening uptake in previous studies, such as deprivation (measured by employment, car ownership, and accommodation arrangement) and distance to screening locations (Maheswaran et al., 2006). The latter is often viewed as a part of broader spatial or geographic accessibility issue (Neutens, 2015), which this paper sought to focus on. There are some empirical evidence on the effect of spatial accessibility on screening. For instance Dai (2010) found that living far to the clinics would discourage women to attend mammography screening in Detroit, USA. However it is interesting to note that once other socio-economic factors were controlled for, such as median income, geographic access would become less statistically significant or insignificant. Similarly, Vallee et al. (2010) found that geographic access measured by density of general practitioners and gynaecologists within an area has little impact on cervical screening overall after average income was adjusted in France. Focusing on colorectal cancer screening in the USA, Mobley et al. (2010) found better geographic access measured by distance to closest facility is associated with poorer screening in 12 states while improved screening in 19 states, after adjusting area-wide deprivation. Thus their results are mixed. A recent study by Henry et al. (2013) found that geographic access which was measured by both the number of mammography facilities and travel time was not associated with late-stage diagnosis after adjusting deprivation in 10 states in the USA. Their study did not look at the effect of car ownership, and thus it is probably the access to cars play an important role, considering there is usually strong correlation between car ownership and deprivation.

There are also evidence on the impact of geographic access from other screening types other than cancer. For instance, Cullinan et al. (2012) found that increased travel distance to screening hospital site could reduce screening uptake rates for gestational diabetes mellitus in Ireland, even deprivation has been adjusted. This finding is however not fully consistent with other studies on cancer screening (which requires long term commitment), as most previous studies discussed above seem to suggest that geographic access measured by travel distance or time has little impact on screening attendance once other socio-economic factors such as deprivation were controlled for. This indicates that, for cancer screenings, it may not be the travel time or distance in itself but other factors that play a role in screening uptake.

Indeed, most previous studies on geographic access and health care rely on travel time or distance based method to measure geographic access (Neutens, 2015). However, geographic access in general does not only concern travel time or distance. From transport point of view, mode of travel, i.e. how people travel, should also be considered, as it can affect the travel experience from one spatial location to another. For example, as argued by Neutens (2015), a person taking public transport for long commuting should not necessarily be considered willing to travel in long distance to explore health care opportunities if the person does not have access to a car in rural areas. This suggests that it is important to consider transport modes that are available to people when one looks at geographic access to health care facilities, such as cancer screening. Car ownership is typically correlated with level of deprivation, and considering the fact that previous studies often found geographic access has little effect on cancer screening after accounting for deprivation and even health insurance status, transport mode availability can be an important factor.

There however seems to be a dearth of literature on the effect of different transport modes on the cancer screening uptake. In particular, how access to private cars and public transport may play a role in cancer screening coverage is less studied. One exception is a study by Woolley et al. (2007) who reported that car accounts for around 59.9–75.4% among those who attend cervical screening while bus only accounts for 7.1–15.1% in parts of the United Kingdom. However, such and similar studies (e.g. Frew et al. (1999), who reported that 80.5% travelled by car, followed by 9.2% by bus) primarily focus on estimating the *costs* of transport among those *attendees*; and while costs may indeed have an impact, it does not offer further insights on which transport mode (e.g. car, bus) is preferred by those who were invited to attend screening, and subsequently how the choice/availability of different transport modes can affect cancer screening uptake. Coughlin and King (2010) looked at the impact of commuting time to work as well as the use of public transport on breast and cervical cancer screening, at the county-level in the USA. They found that no significant association is observed between breast/cervical cancer screening and either the use of public transport or access to a car. However, the transport and health settings in the USA may be considerably different than Europe, and there seems to have been limited evidence from Europe. With the exception such as Coughlin and King (2010), previous studies are mainly based on individual level data, and as such they did not control for area level “system-wide effects”. An aggregate area-wide analysis is vital to understand what factors are associated screening uptake rate, partially because it could be difficult to obtain detailed data from those women who ignore the invitation letters in the case of an individual level analysis. In addition, an area-wide analysis enable us to examine the spatial pattern across the whole country, instead of having to focus on groups of people from a limited number of areas as often in the previous individual-level analyses due to higher cost.

An area-wide analysis is also essential to avoid the atomistic fallacy which refers to the fallacy of drawing inferences at aggregate level based on individual level data (Diez-Roux, 1998). For example, a person's travel behaviour does not only depend on the characteristics of the individual, but also on the culture and general travel behaviour of the local community (e.g. carpooling, use of services such as Uber so a person could travel in a private car they do not own, local people's general attitude towards screening), local crime rate, and relevant transport and health care policies in a local authority. Also regional differences (e.g. London vs. other regions in England) may also have an impact and should be controlled for. Such complex spatial variations could be controlled for by a spatial analysis using an aggregate area-wide level data. Finally, previous studies are also typically based on data with relatively small scale, in terms of number of participants and locations, and as such sample bias may occur.

The objective of this paper is to explore the impact of car ownership and public transport on cancer screening uptake by employing a spatial analysis within England, while controlling for ethnicity, age profile and other relevant socio-economic factors. It is believed that this paper contributes to the literature in the broad area of transport modes and health care access which tends to be less studied. It adds to the debate regarding what role car or public transport has in public health. The rest of the paper is organised as follows: firstly, the data and statistical methods are described; it is then followed by the modelling results and discussion. Finally, conclusion is drawn and future research direction is offered.

## 2 Materials and methods

### 2.1 Data description

The study area covers all district-level areas (e.g. districts, London Boroughs, unitary authorities) in England. Data on cancer screening coverage and related socio-economic factors are made available at district level. There are currently 326 districts in England, with population ranging from 2203 (Isles of Scilly) to more than a million (Birmingham) according to the Census 2011 data.

**Fig. 1:**
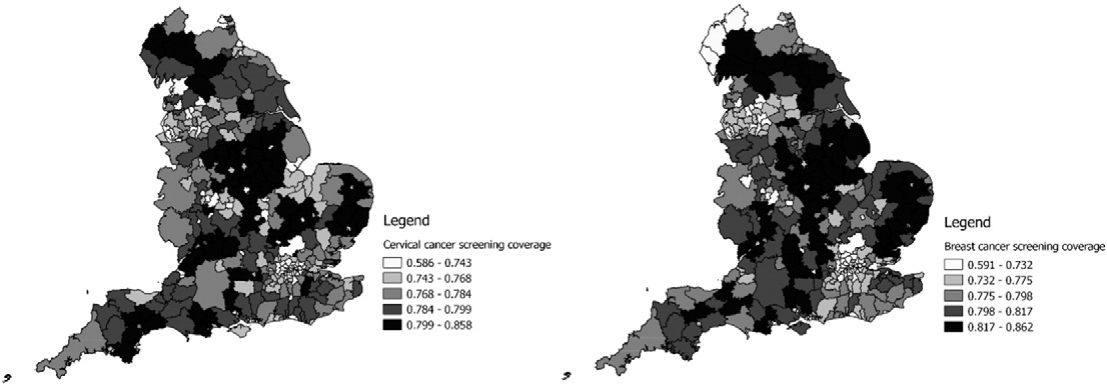
Spatial distribution of breast and cervical cancer screening coverage in England.

There are primarily two sources of data employed in this study. The UK government publishes data on key public health indicators through Public Health Outcomes Framework (2014). The data obtained include the coverage (take-up rate) of cervical and breast cancers, which are the main subjects to be examined in this paper. Women who are registered with a general practitioner (GP) are invited to attend screening in their local screening unit. For breast cancer, women are typically screened every three years; and for cervical cancer, they are screened every three or five years depending on their age in England. Screening coverage is measured by the proportion of people in an area eligible for screening and are screened adequately. In order to ensure the data are consistent with other sources of data such as UK Census 2011 as described below, cervical and breast cancer coverage data for the year 2011 have been used. In addition to cancer screening coverage, fuel poverty (measured by “the percentage of households in an area that experience fuel poverty” – a household was defined as fuel poor where they are on “low income” but require “high costs” of fuel) has also been extracted and controlled for in the following analysis. Fig. 1 shows the spatial distribution of breast and cervical cancer screening coverage in England:

Car ownership, public transport usage, and other relevant socio-economic factors that may affect the cancer screening uptake are obtained from the *UK Census 2011*. The census data contain various useful socio-economic data, such as household car ownership, the usual transport mode for travel to work, economic activity, ethnicity, age, and level of deprivation. These socio-economic factors are hypothesised as potential influencing confounding factors on cancer screening uptake. In the census, a household is defined as “deprived” if they meet one of the following characteristics: employment (any member of a household, who is not a full-time student, is either unemployed or long-term sick); education (low qualification and no person aged 16–18 is a full-time student); health and disability (any person in the household has general health that is ‘bad’ or ‘very bad’ or has a long term health problem); and housing (overcrowded or no central heating). A person aged 16 to 74 is considered as economic active if the person was working or looking for work in the week before census. The proportion of people travelling to work by public transport (i.e. underground/metro/light rail/tram, train, bus/minibus/coach - the census data contains mode share for each district) is commonly used as a proxy for public transport usage in the literature (e.g. Wang et al. (2014)), and as such it has been employed in this paper. However it should be noted that consequently travel information regarding some minority of women, e.g. young women and those who are not employed or work at home, has not been covered in this variable. This may less be an issue in this study since those who are invited for cancer screening are required to be 25–70 years old. In addition, travelling to cancer screening may be different to travelling to other activities such as work. Therefore using this variable to measure public transport usage for cancer screening has its limitations. Although such limitation is due to the nature of linking different data sources, it is believed that this variable is a good proxy for public transport usage and provision in an area (for example London has substantially higher average score at 51.8% than other regions of England ranging from 5.9%–13.7%), and as such may be a factor for travel behaviour including trips to cancer screenings. Finally, proportion of white measures the proportion of people who are ethnically white in an area.

As explained in the earlier section, both sources of data are aggregated at English district level. The two sources of the data are linked using the unique area code for each area. This results in a total number of 320 observations (i.e. districts) in the final dataset after removing the missing data. The average population size in the districts included in the analysis is 162,573. Table 1 shows the summary statistics of data used in this paper.

As can be seen, both breast and cervical cancer screening coverage range from around 60% to 86%, with average at around 77%. Furthermore, in order to account for regional differences, a series of dummy variables have been added with each dummy variable representing a region (e.g. London, East Midlands).

**Fig. 2:**
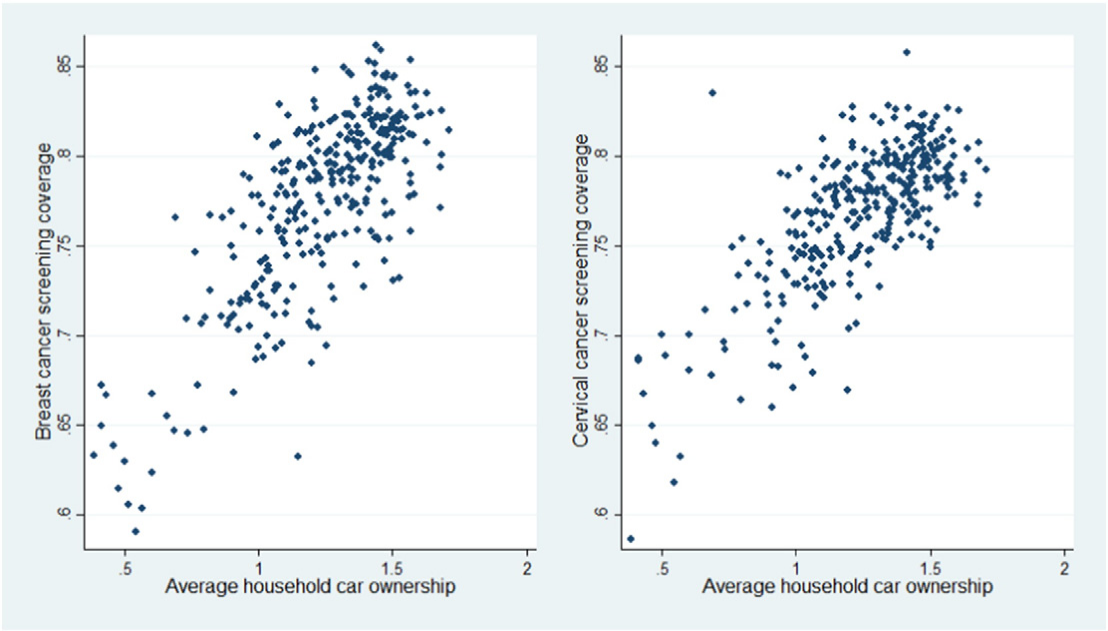
Relationship between cancer screening coverage and household car ownership.

**Fig. 3:**
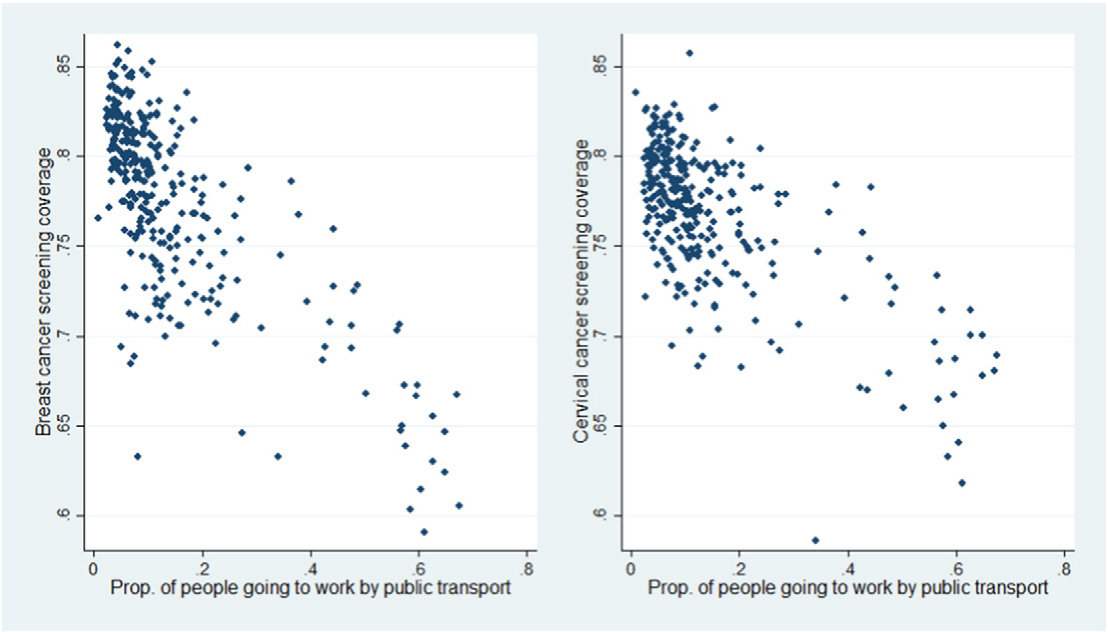
Relationship between cancer screening coverage and public transport usage.

The relationships between cancer screening coverage and various socio-economic factors at the district level were firstly explored using simple scatter plots. Fig. 2, for example, presented such relationship for average household car ownership. As can be seen clearly, there is a positive association between car ownership and breast or cervical cancer screening coverage. The relationship between public transport usage and screening coverage shown in Fig. 3 however, is less linear, though a general inverse relationship can be observed.

Correlations between the social-economic factors have also been explored, and the correlation coefficient between any pair of factors is less than 0.8, suggesting the multicollinearity may not be a serious issue in our data. For example, one may speculate fuel poverty is highly correlated with household deprivation defined in census; however the correlation coefficient between them is only 0.35. In addition, variance inflation factors (VIF) for the various socio-economic variables have been examined and presented in Table 2. Based on the guideline suggested by Chatterjee and Hadi (2012), our data clearly does not exhibit any serious collinearity problem, in particular, between car ownership and deprivation; and hence household income, though unobserved, is unlikely to affect the modelling results. This may be due to the nature of complex relationship between car ownership and income (Dargay, 2007). From a spatial point of view, variations in area characteristics can also affect car ownership. For instance, London has considerably low car ownership (0.60) compared to the rest of England (ranges from 0.91 in North East to 1.35 in South East).

Another concern is that women in a district could go to a screening unit in another district, potentially causing the spillover effects in the response variable. While such cases may be relatively minor, since patients need to live within a GP's catchment area to register in 2011 and the size of a district is relatively large, our spatial model is useful since it explicitly account for such spillover effects in response variable. The statistical methods have been detailed below.

### 2.2 Statistical method

Cancer screening coverage is in the form of proportions, i.e. ranges from 0 to 1, and therefore, regular linear regression model is not suitable for such data (Papke and Wooldridge, 1996). To overcome this, the dependent variable is transformed in such a way as below:

$$y=\log\left(\frac{p}{1-p}\right)$$

where *p* is the cancer screening coverage rate in proportions in an area. A multiple regression is then fitted:

y=Xβ

where ***X*** denotes explanatory variables, such as car ownership and public transport usage; and ***β*** are corresponding coefficients. This model can be estimated using the ordinary least squares (OLS) method.

A common concern for such modelling approach is that the model above ignores spatial correlation which may lead to misleading model estimation results (LeSage, 1999). Observations from one spatial unit may be correlated to observations from near spatial units since they are likely to share similar socio-economic, infrastructure or other characteristics.

To explore whether spatial correlation exists in our data, Moran's I statistics can be calculated, and if spatial correlation does exist, an appropriate model that can control for spatial correlation should be used. There are two classic spatial models depending on where the spatial correlated effects occur: 1) spatial autoregressive (SAR) model, and 2) SAR error model, or simply as spatial error model (SEM) (Anselin, 1988). The SAR model which assumes spatial correlation occurs at dependent variable takes the form:

y=λWy+Xβ+ε

where ***Wy*** is a spatially lagged dependent variable for spatial weights matrix ***W***; *λ* is the scalar for spatial lag coefficient; and ***ε*** is error term which is independent and identically distributed. The ***W*** used here is an inverse-distance spatial-weighting matrix based on centroid locations of districts, i.e. the (s, t)th element of ***W*** is 1/*d*_*st*_, where *d*_*st*_ is the distance between centroids of district s and t. The spatial lag term ***Wy*** can be considered as a spatially weighted average of the dependent variable at neighbouring spatial units.

Alternatively, one may assume that spatial correlation occurs at the error component, which forms a spatial error model:

y=Xβ+u

u=ρWu+ε

where ***u*** is the error term expressing spatial dependence and *ρ* is the spatial autoregressive coefficient. All other terms are as previously defined.

The spatial models are estimated using maximum likelihood method using a Stata package “spreg” developed by Drukker et al. (2013). For model comparison and selection, Akaike information criterion (AIC) can be used to assess goodness-of-fit and complexity between different models. The AIC is defined as: AIC = − 2log*L* + 2*P*, where *L* is the likelihood of the model and *P* is the number of parameters to be estimated in the model. A model with lower AIC value is preferred.

## 3 Modelling results

### 3.1 Non-spatial model

As discussed, an OLS model has been firstly used to explore the relationship between cancer screening coverage and various socio-economic factors. The modelling results for both breast and cervical cancer screening coverage are presented in Table 3.

As can be seen in Table 3, car ownership and public transport usage play an important role in breast cancer screening; while only car ownership is found to affect cervical cancer screening. The exponential form of the coefficients indicates odds ratio. The results thus suggest that if the average household car ownership increases by one unit, the odds of taking up the breast cancer screening would increase by a factor of 1.51, holding all other variables constant. The effect for cervical cancer is relatively smaller, at 1.34.

As for public transport usage, it is found to have a statistically insignificant effect on cervical cancer screening coverage; however, it is found to have a significant inverse association with breast cancer screening coverage, which seems surprising at the first glance. This result suggests better public transport provision is associated with worse breast cancer uptake rate. One may expect that better public transport may improve the screening coverage however conversely this may mean car usage were discouraged so people tend to travel by public transport as a result. This result suggests that women to a great extent are inclined to use cars to attend breast cancer screenings as indicated by both modelling results and previous studies.

Better public transport provision may be achieved at a expense of higher cost of car usage (and hence affecting car ownership), for example less parking spaces, increased parking charges, increased congestion due to less road space allocated to cars, and congestion charges. Indeed, if the variable “prop. of people going to work by public transport” was replaced by “prop. of people going to work by car”, then this variable would become positive and statistically significant. The proportion of travelling by car was not used in the final model as it is highly correlated with car ownership (correlation coefficient: 0.76) which may impose a multicollinearity problem (the correlation coefficient between proportion of travelling by public transport and car ownership is 0.65). This seems to confirm that people would strongly prefer to travel to screening by car, and as such other policies that discourage car usage may reduce screening attendance, even if this means better public transport provision. Another report from Greater Manchester (Threlfall and Fazil, 2009) also noted that “the provision of free transport was ineffective and under-utilised” for breast and cervical cancer screening. This again suggests that private cars may be the most preferable mode of transport for attending screening. A study from the Northern Ireland also found similar behaviour that “more non-attenders did not have access to private transport” (Kee et al., 1992).

In terms of ethnicity, it is found that areas with higher proportion of white had higher prevalence of cervical cancer screening adherence, which is consistent with existing literature (Threlfall and Fazil, 2009). It should be noted that differences in ethnicity may be due to other factors, e.g. deprivation, culture. Similar associations were found for economic activity in an area. As for age, it is found that an area with higher median age is significantly associated with higher screening coverage. This may be due to that older women are more concerned with their health than younger women. Deprivation and fuel poverty are found to have little impact on screening coverage, after adjusting other variables.

Finally, with regard to regional effects, it is interesting to find that women in London are generally more likely to attend breast cancer screening than other regions except East Midlands. Women in London however are less likely to attend cervical cancer screening compared to any other region in England.

### 3.2 Spatial models

As discussed in the statistical method section, ignoring potential spatial correlation may lead to biased estimates, and a spatial model should be used to appropriately account for such spatial effects. Firstly, the Moran's I test which measures similarities and dissimilarities in observations across space was performed. It was found that there are significant spatial correlation at 95% confidence level for both breast and cervical cancers even after controlling for the various socio-economic factors and regional differences. This suggests that a non-spatial model is insufficient and a model which can control for spatial correlation is required.

The modelling results for breast cancer SAR and SEM models are presented in Table 4.

As can be seen, the spatial parameter *λ* in the SAR model for breast cancer coverage is statistically significant at 99% confidence level while the spatial parameter *ρ* in the SEM model is significantly at 99.9% confidence level, indicating that the SAR model may not fully capture spatial correlation compared to the SEM model. The difference in AIC values between SAR and OLS models are very small, confirming that SAR does not improve the non-spatial model much. On the other hand, the AIC values drops considerably from − 363.11 in SAR model to − 406.17 in SEM model. In other words, 12% improvement in AIC value was observed by employing the SEM model, and thus it can be concluded that the SEM is the best model for breast cancer screening data.

Compared to the non-spatial model for breast cancer screening, the results are generally similar except a few variables. For instance, the coefficient of car ownership is 0.41 in non-spatial model and 0.50 in SEM and both are statistically significant. The notable difference is the coefficient of “prop. of households classified as deprived” – while it is insignificant in non-spatial model, it becomes negative and significant at 95% confidence level in SEM. This suggests that if a district has higher proportions of deprived households, the breast cancer screening coverage/uptake would be lower, which is expected. In addition, coefficients for region dummies also changes in SEM, which may be because regional differences are different once spatial correlation has been controlled for.

Similarly, results of spatial models for cervical cancers are estimated and presented in Table 5.

As shown in Table 5, both SAR and SEM have captured the spatial correlated effects as both *λ* in the SAR model and *ρ* in the SEM are statistically significant. The SEM however may be preferred because of better goodness-of-fit in terms of AIC value. Compared to the non-spatial model, the SEM also has a much lower AIC value, suggesting that, again the SEM should be preferred overall. There are also some noticeable differences between SEM and OLS models in terms of coefficients estimates. Public transport usage was previously positive and insignificant but becomes positive and statistically significant in the SEM model, indicating a positive impact of public transport on cervical cancer screening. While this result seems to be inconsistent with the simple exploratory analysis in Fig. 3, our tests showed that confounding caused by both regional differences (for example, London on average has higher public transport usage but lower screening uptake) and spatial correlation contributed to this result. Also, this parameter is significant at 99% confidence level, less than car ownership which is significant at 99.9% level, indicating greater uncertainty on the impact of public transport usage on cervical cancer screening.

Overall, it appears that the spatial model SEM is preferred as it leads to better model in terms of coefficient estimates and goodness-of-fit. This may be due to the fact that spatial correlation is explicitly controlled for in spatial models.

## 4 Discussion

Cancer screening is a useful tool for detection of cancer and thus is important in improving public health. This paper has examined the effects of car ownership and public transport usage on breast and cervical cancer screening coverage in England, along with other relevant socio-economic factors.

Both non-spatial and spatial models (including SAR and SEM) have been employed to analyse breast and cervical cancer screening coverage. There are some differences between non-spatial and spatial models in terms of coefficient estimates, such as the significance level of public transport usage for cervical cancer screening. Spatial models are preferred since they are able to control for spatial correlation which is found to exhibit in the data. The SEM outperforms the OLS and the other classic spatial model SAR in terms of goodness-of-fit. Thus it is recommended to use SEM to model and interpret such type of data.

It is found that car ownership has a positive association with both breast and cervical cancer screening coverage. As for public transport, the results are mixed: it is found that increased public transport usage is associated with reduced breast cancer screening coverage but higher cervical screening coverage. Such differences for the two types of cancers may be due to different targeted population: the targeted age for breast cancer screening is 53 to 70; the targeted age for cervical cancer screening is 25 to 64. The differences in the two age groups may explain the differences in modelling results as women invited for breast cancer screening are generally older than cervical cancer screening. While the finding for the relationship between public transport usage and breast cancer screening seems to be surprising, this may be because journey time by bus is usually considered to be higher than private cars for the same trips. Such more space-time constraint on this group of women using public transport than cars, and together with other perceived benefits of car versus bus, such as convenience and flexibility (Gardner and Abraham, 2007), may ultimately lead to higher preference for cars to travel to screening. Together with the result that car ownership is positively associated with both breast and cervical cancer screening uptake, it may be concluded that increased car ownership and/or higher utility to drive a car than public transport could lead to a better cancer screening coverage in an area. The results thus suggest that people much prefer to use private cars to attend cancer screening than public transport, which echoes previous studies finding car is the dominant mode of transport for attending cancer screening.

Compared to the literature, Coughlin and King (2010) found that generally no important association can be found between breast/cervical cancer screening and either the use of public transport or access to a car in metropolitan areas of United States. While this is not fully consistent with results in this paper, as explained earlier, this may be due to different settings between the USA and England. For instance, it is reported that 68% of women aged 40–64 in the USA had taken a mammogram in the past two years in 2005 (Ward et al., 2008). It seems that women in the USA attend screenings at much younger age compared to the UK. In addition to different populations that screening programs targeted in terms of age in the two countries, financial structures in healthcare are also different that most women screened were on private health insurance in the USA. Whereas in the UK, cancer screening programmes were primarily carried out by the National Health Service (NHS). Thus it could be the case that deprivation may have a larger effect on screening uptake in the USA.

Coughlin and King (2010) however did notice that women in counties with better access to a car were somewhat more likely to have a Pap test, which partially confirms the evidence presented in this paper which also found positive association between car ownership and cervical cancer screening.

It is worth noting that this result is based on an observational study, and thus it does not necessarily imply causal relationships. It is possible that transport and cancer screening are indirectly linked through other unobserved confounding factors, such as their life styles. However, this is the nature of an observational study; and other study design that takes into account causality such as randomized controlled trial is not always feasible in this type of setting, due to various concerns such as ethnicity and cost.

Given such findings on the impact of car ownership and public transport usage, one may advocate better access to private cars. This however may not be compatible with current transport policies in England, which generally promote public transport and walking/cycling, considering various environmental implication of private cars, such as air pollution, road casualties, and congestion. For example, as detailed by Milne (2012), promoting public transport and discouraging cars has the benefit of improved physical activity, reduced road casualties, and reduced air and noise pollution. Therefore some policies advocate heavier tax on cars and road usage (such as congestion charging), less parking spaces, and even “ban car usage” (Milne, 2012). In the light of the empirical evidence found in this study, it is essential that policy makers take very careful consideration of the consequences that these policy interventions would bring. While discouraging cars may lead to some benefit in public health, it may also result in problems in terms of access to health care such as reduced cancer screening uptake. Therefore a balanced policy should be considered, for example providing free parking and/or reduced congestion charge for those attending cancer screening appointments.

Alternatively other changes must be made in public health and transport policy to meet the patients' need, such as decentralisation of cancer services (e.g. mobile screening services), the use of alternative transport arrangements other than traditional public transport or private cars. For the latter, the demand responsive transport (DRT, also known as paratransit) may have its potential as it is usually provided by low capacity road vehicles such as small buses and has no fixed route and/or timetable so can respond to changes in demand (Wang et al., 2014). Although DRT is a form of public transport, it shares many similarities to private cars due to its flexibility and convenience, and thus has the potential for improving screening uptake, especially in the rural areas. Technology advances have also enabled a range of new options, such as Uber, arguably a new form of DRT. It is interesting to see how effective such transport modes can help in health care.

The result from this paper is expected to be informative to policy makers to devise relevant policies to target specific population segment, so as to improve screening coverage, such as young and/or deprived group of people. Also it is interesting that there are some regional differences even after controlling for various socio-economic factors. For example, breast cancer screening in London is generally better than other regions in England. It is yet to examine what has caused this and how it can be improved in such regions.

It is also worth to note that both breast and cervical cancer screenings in this study targeted women. It would be interesting to look at some male focused cancer screenings, such as prostate cancer, so as to compare with breast and cervical cancers and see whether there may be gender differences in terms of transport usage on screening. The *Public Health Outcomes Framework* data used in this study does not cover this type of cancer and it is certainly an area to be further explored.

There are some limitations in the study. It is fair to say that there are other potential barriers to cancer screening and they may have not been taken into account in this study, thus further research is required to control for a wider range of factors. In addition, this study, as with many other spatial aggregate area-wide level studies, may suffer from the modifiable areal unit problem (Neutens, 2015, Openshaw, 1984). To affirm how travel behaviour and transport mode preference is related to cancer screening, the natural next step in research would be to investigate this issue using lower level spatial units, such as English wards. It is also interesting to extend the study to other areas, such as Scotland or other countries, to seek more empirical evidence.

## 5 Conclusion

Household car ownership is found to be positively associated with breast and cervical cancer screening coverage; while the impact of public transport usage is mixed, according to data in England. It is important to control for spatial correlated effects so as to obtain correct parameter estimates in screening coverage models. A balanced transport and public health policy is required to achieve the best health outcome.

## Figures and Tables

**Table 1:** Summary statistics of district-level census and public health data.

Variable	Mean	Std. dev.	Min	Max
Breast cancer screening coverage	0.776	0.052	0.591	0.862
Cervical cancer screening coverage	0.768	0.040	0.586	0.858
Household car ownership (average number of cars or vans per household)	1.238	0.262	0.386	1.710
Proportion of people going to work by public transport	0.142	0.143	0.009	0.675
Proportion of people who are economically active	0.705	0.035	0.608	0.821
Proportion of white	0.892	0.130	0.288	0.989
Median age	40.416	4.458	29	51
Proportion of households classified as deprived	0.562	0.063	0.406	0.750
Fuel poverty	10.645	2.528	2.51	17.97

**Table 2:** Variance inflation factors (VIF) for the various socio-economic variables.

Variable	VIF
Household car ownership	5.38
Prop. of white	4.89
Prop. of people going to work by public transport	4.60
Median age	3.69
Prop. of households classified as deprived	3.47
Prop. of people who are economically active	2.39
Fuel poverty	1.56

**Table 3:** Results from non-spatial models for breast and cervical cancer screening coverage.

	Breast cancer		Cervical cancer	
	Coef.	t-Stat	Coef.	t-Stat
Household car ownership	0.41⁎⁎⁎	5.59	0.29⁎⁎⁎	4.61
Prop. of people going to work by public transport	− 0.81⁎⁎⁎	− 4.93	0.14	1.01
Prop. of people who are economically active	− 0.44	− 1.18	0.76⁎	2.37
Prop. of white	0.19	1.42	0.29⁎	2.47
Median age	0.01⁎	2.14	0.01⁎⁎⁎	4.69
Prop. of households classified as deprived	− 0.38	− 1.63	0.06	0.29
Fuel poverty	− 0.003	− 0.61	0.0005	0.11
Regions				
London	Reference case			
East Midlands	0.08	1.13	0.25⁎⁎⁎	4.17
East of England	− 0.11	− 1.72	0.12⁎	2.17
North East	− 0.01	− 0.14	0.16⁎	2.47
North West	− 0.26⁎⁎⁎	− 3.80	0.10	1.70
South East	− 0.16⁎⁎	− 2.66	0.11⁎	2.14
South West	− 0.14⁎	− 2.06	0.17⁎⁎	2.83
West Midlands	− 0.13	− 1.82	0.10	1.68
Yorkshire and the Humber	− 0.01	− 0.17	0.23⁎⁎⁎	3.69
Constant	1.05⁎	2.3	− 0.72	− 1.82
Statistics				
N	320		320	
R-squared	0.79		0.72	
AIC	− 359.56		− 450.48	

⁎ p < 0.05.

⁎⁎ p < 0.01.

⁎⁎⁎ p < 0.001.

**Table 4:** Spatial models for breast cancer.

	SAR		SEM	
	Coef.	z-Value	Coef.	z-Value
Household car ownership	0.46⁎⁎⁎	6.33	0.50⁎⁎⁎	6.05
Prop. of people going to work by public transport	− 0.61⁎⁎⁎	− 3.54	− 0.84⁎⁎⁎	− 5.09
Prop. of people who are economically active	− 0.42	− 1.18	− 0.16	− 0.48
Prop. of white	0.13	0.94	0.09	0.66
Median age	0.01	1.42	0.01⁎	2.02
Prop. of households classified as deprived	− 0.45⁎	− 1.96	− 0.48⁎	− 2.00
Fuel poverty	− 0.01	− 1.31	0.004	0.82
Regions				
London	Reference case			
East Midlands	0.12	1.75	− 0.002	− 0.02
East of England	− 0.11	− 1.84	− 0.13⁎	− 2.11
North East	− 0.06	− 0.74	0.05	0.65
North West	− 0.26⁎⁎⁎	− 3.98	− 0.22⁎⁎	− 2.9
South East	− 0.17⁎⁎	− 2.88	− 0.07	− 1.19
South West	− 0.18⁎⁎	− 2.6	− 0.1	− 1.52
West Midlands	− 0.10	− 1.41	− 0.20⁎⁎	− 2.61
Yorkshire and the Humber	− 0.03	− 0.44	0.03	0.37
Constant	1.36⁎⁎	3.00	0.82	1.89
*λ*	− 48.52⁎⁎	− 2.76	–	
*ρ*	–		846.98⁎⁎⁎	15.46
Statistics				
N	320		320	
Log-likelihood	199.56		221.09	
AIC	− 363.11		− 406.17	

⁎ p < 0.05.

⁎⁎ p < 0.01.

⁎⁎⁎ p < 0.001.

**Table 5:** Spatial models for cervical cancer.

	SAR		SEM	
	Coef.	z-Value	Coef.	z-Value
Household car ownership	0.35⁎⁎⁎	5.58	0.22⁎⁎⁎	3.42
Prop. of people going to work by public transport	0.39⁎	2.56	0.42⁎⁎	2.92
Prop. of people who are economically active	0.78⁎	2.57	1.07⁎⁎⁎	3.85
Prop. of white	0.22	1.93	0.22⁎	2.06
Median age	0.01⁎⁎⁎	3.91	0.02⁎⁎⁎	7.00
Prop. of households classified as deprived	− 0.01	− 0.06	− 0.19	− 0.98
Fuel poverty	− 0.004	− 0.84	− 0.002	− 0.45
Regions				
London	Reference case			
East Midlands	0.29⁎⁎⁎	4.93	0.02	0.32
East of England	0.11⁎	2.10	0.02	0.44
North East	0.11	1.65	0.03	0.51
North West	0.09	1.70	− 0.09	− 1.41
South East	0.10⁎	2.01	0.04	0.73
South West	0.13⁎	2.20	0.06	1.00
West Midlands	0.13⁎	2.21	− 0.10	− 1.53
Yorkshire and the Humber	0.20⁎⁎⁎	3.40	0.03	0.55
Constant	− 0.39	− 1.00	− 0.83⁎	− 2.33
*λ*	− 55.35⁎⁎⁎	− 3.64		
*ρ*			550.47⁎⁎⁎	30.75
Statistics				
N	320		320	
Log-likelihood	247.75		278.91	
AIC	− 459.49		− 521.81	

⁎ p < 0.05.

⁎⁎ p < 0.01.

⁎⁎⁎ p < 0.001.
